# Estimation of the Potential Infestation Area of Newly-invaded Fall Armyworm *Spodoptera Frugiperda* in the Yangtze River Valley of China

**DOI:** 10.3390/insects10090298

**Published:** 2019-09-13

**Authors:** Qiu-Lin Wu, Li-Mei He, Xiu-Jing Shen, Yu-Ying Jiang, Jie Liu, Gao Hu, Kong-Ming Wu

**Affiliations:** 1 State Key Laboratory for Biology of Plant Diseases and Insect Pests, Institute of Plant Protection, Chinese Academy of Agricultural Sciences, Beijing 100193, China; 2 College of Plant Protection, Fujian Agriculture and Forestry University, Fuzhou 350002, China; 3 National Agro-Tech Extension and Service Center, Beijing 100125, China; 4 Department of Entomology, Nanjing Agricultural University, Nanjing 210095, China

**Keywords:** *Spodoptera frugiperda*, exotic pest, windborne migration, atmospheric circulation, trajectory analysis, forecasting

## Abstract

The fall armyworm (FAW), native to the Americas, has rapidly invaded the whole of Southern China since January 2019. In addition, it can survive and breed in the key maize- and rice- growing area of the Yangtze River Valley. Furthermore, this pest is also likely to continue infiltrating other cropping regions in China, where food security is facing a severe threat. To understand the potential infestation area of newly-invaded FAW from the Yangtze River Valley, we simulated and predicted the possible flight pathways and range of the populations using a numerical trajectory modelling method combining meteorological data and self-powered flight behavior parameters of FAW. Our results indicate that the emigration of the first and second generations of newly-invaded FAW initiating from the Yangtze River Valley started on 20 May 2019 and ended on 30 July 2019. The spread of migratory FAW benefitted from transport on the southerly summer monsoon so that FAW emigrants from the Yangtze River Valley can reach northern China. The maize-cropping areas of Northeastern China, the Korean Peninsula and Japan are at a high risk. This study provides a basis for early warning and a broad picture of FAW migration from the Yangtze River Valley.

## 1. Introduction

The fall armyworm (FAW), *Spodoptera frugiperda* (J. E. Smith) (Lepidoptera: Noctuidae), an important crop and forage pest native to the Americas [1,2], is Africa’s and Asia’s “winged invader and farmers’ latest foe” [3,4,5]. In much of North and South America, FAW outbreaks are sporadic but can be severe [2,6]. FAW larvae attack a total of 353 host plant species belonging to 76 plant families [7], and threaten a variety of crops including maize, rice, sugarcane, sorghum, and cotton [2]. Two biotypes, corn strain and rice strain, were identified, distinctly differing in host plant preferences [8,9]. Because it lacks a diapause to survive during winter in temperate zones, FAW overwinters only as far north as the southernmost areas of Texas and Florida [1,2,10]. In the overwintering range in the U.S., the FAW generations are continuous and the total developmental duration is approximately 4 weeks during the summer (June–August), and three months during winter (December to February) [6]. Each spring, tropical-originating FAW undertake a succession of northward migratory flights to re-infest cropping areas in the mid-latitude temperate zone, where it can also breed continuously when the climate is suitable and the host plants are available [11]. By the end of the growing season in North America, Ontario and Québec, Canada, have been recorded as the northernmost regions regularly infested by FAW [12,13]. Conversely, the southward (return) migration of FAW was also supported by the analysis of pheromone trap catch data and associated wind currents [13,14]. The multi-generational and nocturnal long-distance migration of FAW adults benefiting from high-altitude winds has been well demonstrated in North America using multiple techniques, such as large-scale pheromone/light traps, radars, and atmospheric simulation models [15,16,17,18]. 

**Figure 1:**
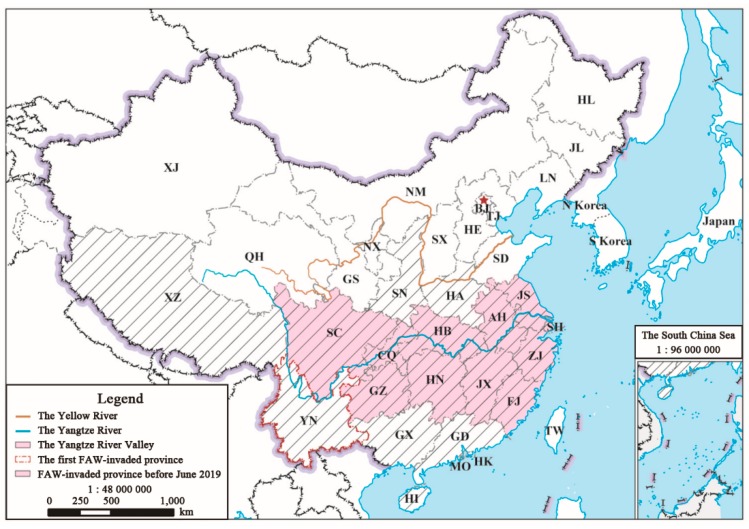
The fall armyworm (FAW) invasion situation before June 2019 and the study area in China. Abbreviations: AH: Anhui, BJ: Beijing, CQ: Chongqing, FJ: Fujian, GD: Guangdong, GS: Gansu, GX: Guangxi, GZ: Guizhou, HA: Henan, HB: Hubei, HE: Hebei, HI: Hainan, HK: Hongkong, HN: Hunan, HL: Heilongjiang, JL: Jilin, JS: Jiangsu, JX: Jiangxi, LN: Liaoning, MO: Macao, NM: Inner Mongolia, NX: Ningxia, SC: Sichuan, SD: Shandong, SH: Shanghai, SX: Shanxi, SN: Shaanxi, TJ: Tianjin, TW: Taiwan, XJ: Xinjiang, XZ: Xizang(Tibet autonomous region), YN: Yunnan and ZJ: Zhejiang.

In early 2016, FAW appeared on maize crops in West Africa [3,19], the first time a population was reported outside of the Americas. The most likely route of invasion to Africa is by air transportation, but subsequent natural migratory flight resulted in FAW colonizing 28 African countries by August 2017 [19]. This pest had caused millions of dollars of losses in Africa [11,20]. As a strong flyer, FAW has continued to spread beyond Africa and was confirmed to be established in India in May 2018 [4]. By January 2019, Yemen [21], Thailand [22], Sri Lanka [23], Bangladesh [24], Myanmar [25], and Yunnan in China [26] all reported being invaded by the FAW population. A previous study indicated that Myanmar is one of the key source regions of FAW immigrants threatening the crop yield in the tropical and southern subtropical zones of China [27], which comprise Yunnan (YN), Guangxi (GX), Guangdong (GD) and Hainan (HI) (Figure 1). At present, this exotic pest has formed a resident population in these zones [28] and invaded up to 17 provinces and one municipality since its first invasion in China in January 2019 [29]. Among 18 regions mentioned above, the Yangtze River Valley, which has been largely colonized by FAW originating from the tropical and southern subtropical China [28], is located in Central China and consists of 11 provinces (including two municipalities): Sichuan (SC), Chongqing (CQ), Guizhou (GZ), Hubei (HB), Hunan (HN), Anhui (AH), Jiangxi (JX), Jiangsu (JS), Zhejiang (ZJ), Fujian (FJ), and Shanghai (SH) (Figure 1). However, FAW invaders are unable to survive the winter in these locations [30]. Based on numerous analyses of migration patterns of insects in China, such as the oriental armyworm *Mythimna separata* [31], cotton bollworm *Helicoverpa armigera* [32], rice leaf folder *Cnaphalocrocis medinalis* [33], and brown planthopper *Nilaparvata lugens* [34], the Yangtze River Valley is also supposed to be essential for both supporting FAW invaders which would colonize the North China in summer and receiving returning migrants from North China in autumn. Furthermore, FAW individuals in China collected from 131 counties and cities of 13 provinces, viz. YN, GX, GD, HI, SC, GZ, HN, HB, FJ, ZJ, JX, CQ and SH, belonged mostly to the corn strain [35].As the annual East Asian summer monsoon can effectively transport migrating insects on its prevailing southerly winds [28,36], FAW developing in the Yangtze River Valley has a huge potential to disperse northward; where the staple crop is maize. Therefore, studying and predicting the possible migratory pathways and distributions of the newly-emerged generations of the early arrivals of FAW in the Yangtze River Valley are critical. This information would also assist in raising awareness, monitoring for early detection, and formulating appropriate management strategies both nationally and regionally [30].

In this study, we collected the first-reported date of FAW appearance for each monitoring site in the Yangtze River Valley by systematic field investigations. As highly active flights of moths occur during dominant periods of emergence [37,38], and air temperature is one of the most important climatic factors in FAW’s development, we then determined the eclosion peak days as the emigration dates of the first and second generations of the newly-invaded FAW populations by analyzing the historical daily mean temperatures. Subsequently, large-scale synoptic patterns in corresponding periods of both the last five years from 2014 to 2018 and 2019 were studied to illustrate and interpret the rapid aerial spread of FAW. Lastly, we simulated and predicted the possible flight pathways of migratory FAW populations taking off from the Yangtze River Valley using a numerical trajectory modelling method combining the self-powered flight behavior parameters (initiation time, flight altitude selection, duration, flight heading, self-powered airspeed) combined with high-resolution meteorological conditions provided by the Weather Research and Forecasting (WRF) model [39]. In addition to the effects of airspeed and orientation on migratory displacement, unfavorable atmospheric factors or phenomena terminating insect flight, such as low temperature [36], were also considered during the simulation process.

## 2. Materials and Methods

### 2.1. Study Area and Field Investigations of Invasive FAW before June 2019 in the Yangtze River Valley

**Figure 2:**
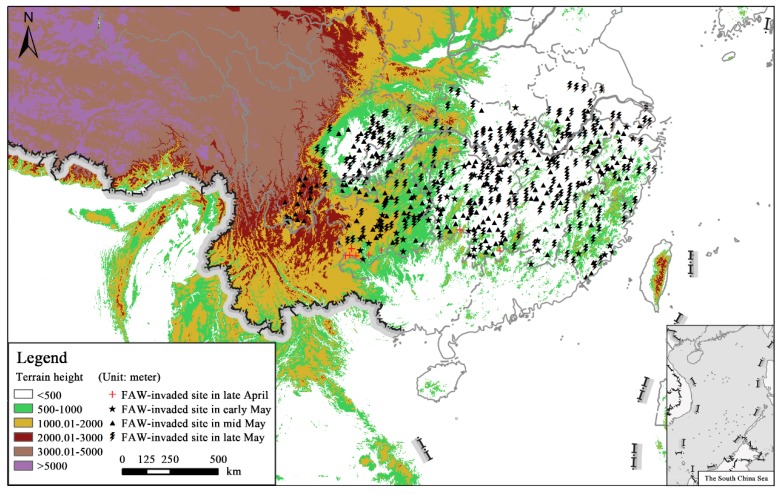
Topographic terrain and field survey locations of the Yangtze River Valley of China.

Map data were derived from the China National Platform for Common GeoSpatial Information Services (National Geomatics Center of China, Beijing, China). The Yangtze River Valley has a diverse range of topography, including mountains, plateaus, basins (tributaries), hills, and plains (Figure 2). In particular, SC, GZ, and CQ are surrounded by the Tibetan Plateau, Hengduan Mountains, Yunnan-Guizhou Plateau, and Sichuan Basin, with an altitude of 200–2000 m, whereas the Lower Yangtze River Valley is < 1000 m. Xinyang City, Henan province (HA, Figure 1) was also included in the present study because this city is near the Yangtze River Valley region.

Systematic field investigations throughout the Yangtze River Valley were conducted by the China National Agro-Tec Extension and Service Center (NATESC) (Beijing, China). Therefore, the first invasion date and the host plants of FAW were recorded.

### 2.2. Estimations of Emigration Periods of the First and Second FAW Generations of Newly-invaded Populations

As the timing of certain life history stages is extremely difficult to be observed directly in the field, we used evidence of the effects of temperature on FAW’s life cycle [40,41] to estimate the emergence period of FAW from the studied region. According to the main life history stage investigated in fields of the Yangtze River Valley, our laboratory experiments indicated that the number of days from fourth instar larvae to eclosion at the constant temperatures of 20 °C and 25 °C were 30 and 20 days, respectively. FAW needs 50 and 30 days to complete a life cycle at these temperatures [42]. This result is consistent with previous literatures related to the effect of temperature on the development of FAW in the Americas [40,41].

We used the daily mean surface air temperature (DMSAT) (Version 3.0; dataset code: SURF_CLI_CHN_MUL_DAY; National Meteorological Information Center, Beijing, P. R. China) in our study. Due to the lack of meteorological data (i.e., temperature, wind, pressure etc.), from June to August 2019, we firstly evaluated the differences between the historical DMSAT from 2014 to 2018 and that of 2019 by comparing the DMSAT of each day from 20 April to 30 May. The analysis was conducted throughout the Yangtze River Valley by a Wilcoxon signed-rank test in JMP Version 13.2.0 software (SAS Institute Inc., Cary, NC, USA). All *p*-values ≤ 0.05 were considered statistically significant.

Before estimating emigration periods of the next generations of newly-invaded populations in the Yangtze River Valley, we calculated the number of generations that can develop in this region from the FAW arrival date of 20 April 2019. The threshold temperature and effective accumulated temperatures for development of FAW were 9.16 °C and 680.02 degree-day, respectively [42]. We extracted the DMSATs from 20 April to 20 July 2014 through 2018, for the Yangtze River Valley. The results show that at least two generations of FAW can survive in the Yangtze River Valley after 20 April, if the host plants are available. Hence, migration trajectories of the first and second generations of the newly-invaded FAW population from the Yangtze River Valley were simulated in this study.

According to the field investigation, the temperature data were arranged in groups A to D with 10-day time intervals. The emigratory timings of the first and second generations of the newly-invaded FAW populations were also grouped and identified based on the mean of the historical DMSAT among groups A to D, named A1, A2, B1, B2, C1, C2, D1 and D2. We used the historical meteorological data from 2014 to 2018 to estimate the emigration periods for the first and second generations of FAW adults, and conducted the trajectory analysis using historical meteorological data.

Based on the evaluation detailed above, the DMSATs of all stations (472 locations in 11 provinces) from 20 April to 20 August (possible longest developmental duration for two generations is 80 days) were extracted from the five-year historical data from 2014 to 2018 by applying the Grid Analysis Display System (GrADS) Version 2.0.1 (COLA, Fairfax, VA, USA). Because stations investigated in nature had fluctuating temperatures, we considered each group from A to D as having a constant temperature of 20 °C if the average value of the DMSATs of all locations in each group was lower than 22.5 °C. Conversely, the group had a constant temperature of 25 °C if the average value of the DMSATs of the grouped locations was more than 22.5 °C. As proposed above, each group had four possibilities for the developmental durations of the first and second generations of newly-invaded FAW adults: 30 and 50 days, 30 and 30 days, 20 and 50 days, and 20 and 30 days. We tested the mean of the DMSATs of each group during each possible developmental duration; thus, the emigration periods for the first and second FAW generations of FAW adults could be reasonably suggested.

### 2.3. Analysis of Migratory Flight Relative to Atmospheric Support

The National Centers for Environmental Prediction (NCEP; http://www.ncep.noaa.gov/) Final Analysis data (FNL) [43] were applied as transporting conditions for the migration of FAW. The FNL data with grid distances of 1° × 1° were operationally exported every 6 h. In the present study, nocturnal wind conditions at 925 hPa (approximately 800 m above ground level (AGL)) were extracted and displayed by adopting GrADS V2.0.1 (COLA, Fairfax, VA, USA) to elucidate associations between wind currents and the FAW migration processes.

### 2.4. Numerical Trajectory Modelling Method

The WRF model, which is a next-generation meso-scale numerical weather research model, has been well descried in previous studies [36,44]. We implemented the WRF model to conduct detailed simulations and predictions of FAW migratory pathways. The 1° × 1° and 6-h FNL data were applied as the initial and boundary conditions for driving the WRF model. We used WRF model simulations to obtain meteorological variable values at 1-h intervals for running a three-dimensional trajectory program written in Fortran Language [45] (China National Copyright of Computer Software No. 2015SR090706, 2015) that calculates FAW meso-scale migratory trajectories. The model domain occupies all study provinces shown in Figure 1. The model scheme and parameterizations were previously listed [27,28].

The possible migratory trajectories of FAW at a meso-scale were calculated using the forward trajectory program, and the calculations were determined by the following assumptions: (1) FAW migrates downwind and its aerial movement is dominated by the wind vector and a constant airspeed of 4.5 m·s^−1^ [17,46,47]; (2) FAW exhibits a common orientation to the right of the downwind vector, and the crab angle (i.e., difference between the insect alignment and wind displacement direction) is 30° [17]; (3) migrants typically take-off at 18:00 the local time, and migrate at around 500 AGL for a flight duration of up to 12 h [13,14,18]; (4) migrants undertake one way and up to three consecutive night flights and can land at any time along the migratory route [18]. For the second/third night flight, trajectory simulation continued from the last calculated locations of the first/second night’s trajectories; and (5) FAW cannot fly at atmospheric temperatures <10.0 °C [48,49]. Specifically, a forward trajectory starting at a given departure location where FAW was detected during the field investigations was calculated from 18:00 the local time for up to 12-h each night during the emigration periods of FAW populations colonizing in the Yangtze River Valley and HA.

The starting flight heights of FAW were set to 500, 750, 1000, and 1250 m above mean sea level (AMSL) at source sites with a flat and broad topography, including HA, JS, SH, ZJ, AH, JX, FJ, HN and HB. In contrast, the AMSL values of the starting flight heights of FAW for sites in mountainous SC, GZ and CQ were set to 1500, 1750, 2000 and 2500 m.

We removed endpoints calculated to land in regions without susceptible maize plants or terminating over the sea (e.g., the Yellow Sea between China and the Korean Peninsula) along the 12-h flight routes. Therefore, the invasion rate (%) could be defined as the ratio of the number of valid endpoints within a specific province or region to the total number of valid endpoints [36].

## 3. Results

### 3.1. Large-scale Invasions of Immigratory FAW in the Yangtze River Valley

Six counties in GZ and two in HN confirmed the colonization of FAW in late April (20–30 April 2019; Figure 2); however, the number of newly-invaded counties abruptly increased to 61 in early May (1–10 May 2019) and seven newly-occupied provinces, viz. CQ (the first report date was 1 May), ZJ (2 May), FJ (6 May), SC (8 May), HB (8 May), JX (8 May), and HA (10 May), were also detected. In mid-May (10–20 May 2019), a total of 168 counties throughout the above-mentioned provinces were attacked by FAW larvae. Corn crops in 40 counties in HN, 29 in SC, 26 in GZ, 21 in JX, 18 in AH, 11 in HB, 11 in ZJ, 7 in CQ, 5 in FJ and 3 in SC were all damaged by FAW. Other 235 counties were checked for the occurrence of invasive FAW in late May (21–30 May 2019), and the dates of the first-reported damage in SH and JS were on 22 May and 24 May, respectively. FAW colonized new habitats in the lower Yangtze River Valley comprising HB, HN, JX, and AH. The newly-invaded distribution of FAW mostly occurred in plains below 500 m, although populations also established in mountainous areas like the Yunnan-Guizhou Plateau. The host plants were all maize, and the FAW detected in the Yangtze River Valley during 20 April to 30 May were mostly IV and V instar caterpillars.

### 3.2. Temperature Dynamics in the Yangtze River Valley During the FAW Invasion Period

**Figure 3:**
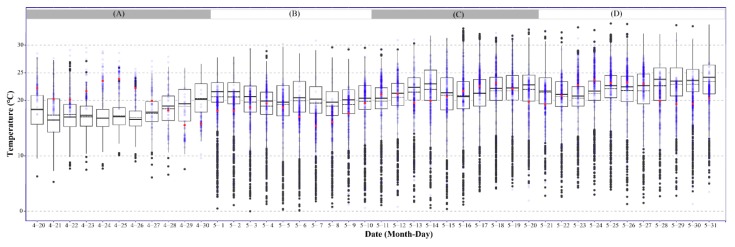
Daily mean surface air temperature in the Yangtze River Valley for 20 April to 31 May of 2014-2018 (black box) and 2019 (blue dot). Based on our field surveys, the Daily Mean Surface Atmospheric Temperature (DMSAT) of late April in two provinces (GZ and HN, grouped by A), early May in 9 provinces (GZ, HN, CQ, ZJ, FJ, SC, HB, JX and HA, grouped by B), of mid-May in 10 provinces (HN, SC, GZ, JX, AH, HB, ZJ, CQ, FJ, and SC, grouped by C), and late May in 11 provinces (HN, GZ, JX, AH, HB, ZJ, CQ, FJ, SC, JS, and SH, grouped by D) were plotted. The bottom and top of the black box indicate the lower and upper quartile values, respectively. The horizonal solid black line shows the median for each category and the black dashed line represents the mean. Whiskers indicate the 5th and 95th percentiles, and the black circle represents the outlier for the data set of 2014–2018. The blue dot represents the observation value in each studied province for 2019, whereas the red circle is the mean for each category.

The regional DMSATs from 2014 to 2018 were investigated to evaluate the change in temperature in FAW-invaded provinces over the Yangtze River Valley in 2019 (Figure 3). The DMSATs for group A to D were separately analyzed (Figure 3). The DMSATs of 2019 in the Yangtze River Valley during 20 April to 31 May were not significantly different from those of the past five years (*n* = 41, Wilcoxon signed-rank test: *p* > 0.1). Therefore, the emigration dynamics of the newly-invaded FAW population in the Yangtze River Valley could be accurately inferred using the historical DMSATs.

**Figure 4:**
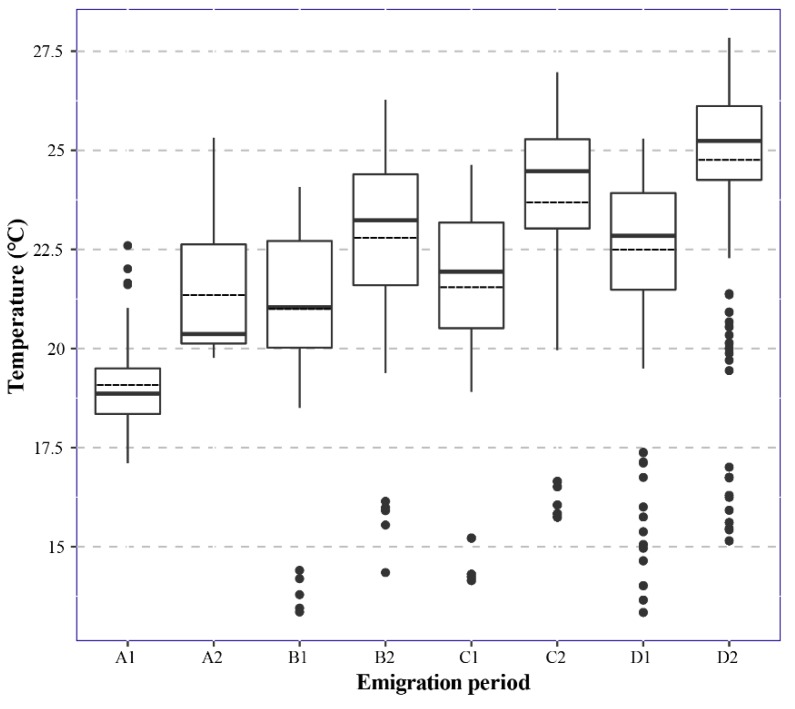
Daily mean surface air temperature for the estimated emigration dates of the first and second generations of newly-emerged adults in the Yangtze River Valley from 30 April to 30 July of 2014–2018. The bottom and top of the black box indicate the lower and upper quartile values, respectively. The horizonal solid black line shows the median for each category, and the black dashed line represents the mean. Whiskers indicate the 5th and 95th percentiles; the black circle represents the outlier for the data set of 2014–2018. Furthermore, A1 and A2 are the first and second generations of newly-emerged adults, respectively, for counties in provinces suffering from invasions of FAW in late April 2019; B1 and B2 are the freshly-infested counties in early May; C1 and C2 are for counties infested in mid-May; and D1 and D2 are for counties in late May.

The averages of the historical DMSATs for both A1 and A2 arriving in the target provinces (GZ and HN), i.e., the first and second generations of the newly-invaded FAW populations during late April, were less than 22.5 °C during two successive periods of 20 April to 30 May and 31 May to 30 July (Figure 4); the average DMSATs for B1 was also lower than 22.5 °C during 1 May to 10 June, whereas the value for B2 from 11 June to 10 July in their target provinces was 22.80 °C, the value was 21.55 °C for C1 from 11 May to 20 June, and 23.69 °C for C2 from 21 June to 20 July. The temperature increased during July, and the averages DMSATs were 22.49 °C and 24.48 °C for D1 during the time period from 21 May to 30 June and D2 from 1 July to 30 July, respectively. We concluded that the temperature from 20 April to 30 July was suitable for the development of the invasive FAW.

### 3.3. Migration Arena

The migration activities of FAW taking-off from the Yangtze River Valley would mostly start from 20 May and last until 30 July. Specifically, the migration peak of the first next-generation adults was between 20 May and 30 June, and the peak of the second next-generation adults was from 1 July to 30 July.

**Figure 5:**
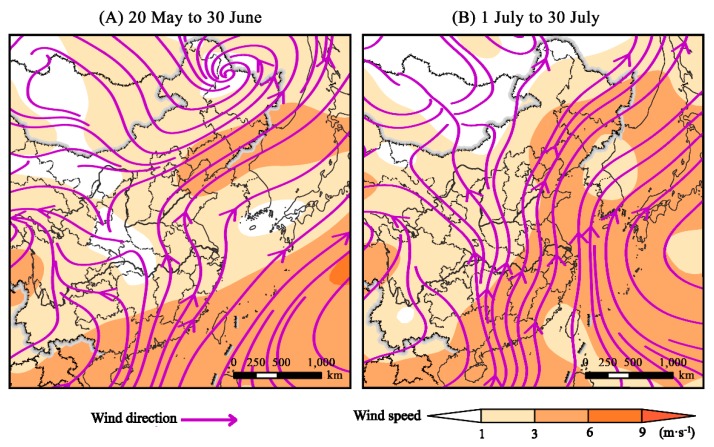
Average night wind fields at 925 hPa from 20 May to 30 June (**A**) and from 1 July to 30 July (**B**) in East China responsible for the migration processes of the first and second generations of the newly-invaded FAW populations in the Yangtze River Valley of China.

The nocturnal wind currents on the estimated emigration days in 2014–2018 blew to the north during the emigration periods over the whole region of East China (Figure 5), which is a key agricultural cropping area. Therefore, FAW migrants already in flight can be efficiently carried northward by the prevailing southerly airstreams. From 20 May to 30 June, easterly winds also occurred in the middle Yangtze River Valley, which may encourage newly-emerged adults to proceed westwards (Figure 5A). In particular, winds were much stronger in July with wind speeds varying from 3 to 6 m·s^–1^ than those moving across the East Asia during 20 May to 30 June and greater than the airspeed of FAW migrants at 4.5 m·s^−1^ (Figure 5B).

### 3.4. Simulated Flight Trajectories, Landing Sites and Distribution Regions

**Figure 6:**
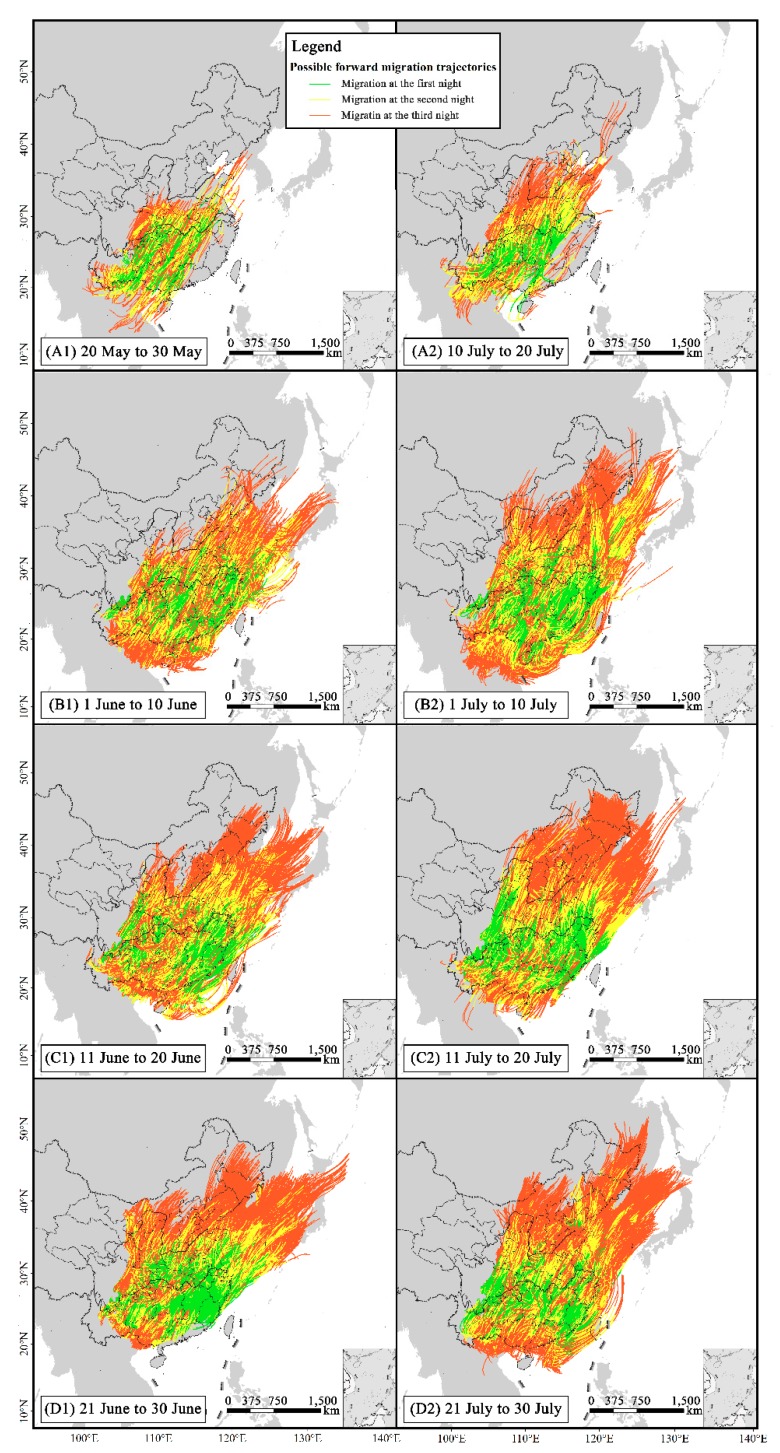
Migration trajectories of the first (**left**) and second (**right**) generations of the newly-invaded FAW populations from the Yangtze River Valley of China. **A1** and **A2** are the migration trajectories of the first and second generations of newly-emerged adults during the peak periods of emigrations, respectively, for the counties in provinces suffering from invasions of FAW in late April 2019; **B1** and **B2** for the freshly-infested counties in early May; **C1** and **C2** for the freshly-infested counties in mid-May; and **D1** and **D2** for the freshly-infested counties in late May.

**Figure 7:**
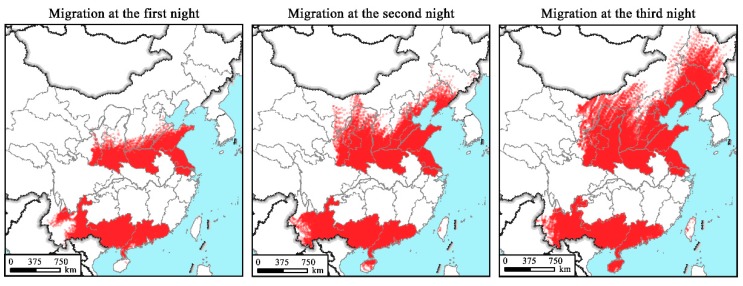
Landing sites and distribution regions of the first generations of the newly-invaded FAW populations from the Yangtze River Valley of China. Red dots indicate the endpoints of forward trajectories.

**Figure 8:**
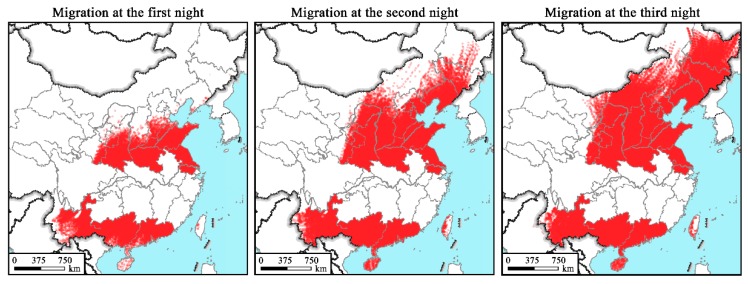
Landing sites and distribution regions of the second generations of the newly-invaded FAW populations from the Yangtze River Valley of China. Red dots indicate the endpoints of forward trajectories.

The wind fields resulted in the spread of the downwind FAW emigrants during the studied periods from 20 May to 30 July (Figure 6). In particular, the estimated first generation of newly-invaded FAW emerged on 20–30 May for A1, on 1–10 June for B1, on 11–20 June for C1, and on 21–30 June for D1, whereas the peak adult emergence of the second generation was suggested to occur on 20–30 May for A1, 1–10 June for B1, 11–20 June for C1, and 21–30 June for D1, repectively. The first generation of FAW from the Yangtze River Valley primarily migrated northeastward to maize production areas in East China arriving there by late June (Figure 6(A1, B1, C1, D1)). As presented in Figure 7, migrations initiating before 10 June mostly fly within the southern parts of the Yellow River. The first generation of FAW initiating their flights from the Yangtze River Valley can also spread into Northeastern China, the Korean Peninsula (Figure 6(A1, B1, C1, D1) and Figure 7), and as far as Japan, if they undertake two or three successive night flights (Figure 6(B1, C1, D1)). Simulated FAW migration of the second generation from the Yangtze River Valley would occur in July and spread northeastward across East China into Northeastern China and the Korean Peninsula (Figure 6(A2, B2, C2, D2)). There are substantial northern landing endpoints beyond the Yellow River and those of the first-night travel covering much of northern China including the provinces of SD, HA, JS and Shaanxi (SN) (Figure 8).

In total, 684,960 and 924,055 endpoints of the first (Figure 6(A1, B1, C1, D1)) and second (Figure 6(A2, B2, C2, D2)) simulated emigration waves were obtained, respectively. For both emigration waves, the trajectory endpoints were largely concentrated in the provinces of SN (8.59% and 12.72% for the first and second waves, respectively), SD (10.50% and 12.56%), and HA (15.59% and 15.27%). JS, which had very few invaded locations, was also included as the damaged destination in this study, where the proportions of the first and second simulated emigration waves were 18.89% and 13.33%, respectively (Figure 7 and Figure 8).

The tropical and southern subtropical zones of China including YN, GX, GD and HN (Figure 1), containing 32.40% and 22.02% of all calculated endpoints for first and second migration waves, respectively, were also destinations of FAW from the Yangtze River Valley (Figure 7 and Figure 8). Of all the endpoints, 8.33% were located in the Korean Peninsula. Gansu (GS), Liaoning (LN), Hebing (HE) and Shanxi (SX) were also under the threat of FAW invasion with total invasion rates of 3–5%.

## 4. Discussion

As of late April and May 2019, the presence of newly-invaded FAW feeding on maize plants was confirmed in all 472 studied counties in the Yangtze River Valley (Figure 2). Our results are consistent with the analysis of molecular characteristics of FAW individuals sampled from 131 counties throughout the south of China, including FJ, GZ, HB, HN, JX, SC, SH, ZJ and CQ in the Yangtze River Valley, which proves that the FAW primarily belonged to the corn strain [35].

However, according to the most recent personal observations by our research group, FAW larvae were also able to attack sugarcane and sorghum in YN, GX, HI, GZ and CQ in China [50]. FAW damage to sugarcane was also reported in Maharashtra, India [51]. In Particular, India has reported the detection of the rice strain in the state of Karnataka [52]. Different genotypes of FAW in China have also been discovered [35,53]; thus, the FAW populations infesting China are suspected to originate from offspring of the hybrid population of both the corn and rice strains [35], which suggests that regions growing rice, like the middle and lower Yangtze River Valley, should be aware of the potential risk from FAW.

Previous studies illustrated a strong correlation between the numbers emerging each night and the peak volume density measured by radar during the following evening [37,38]; thus, the accuracy of calculation of the FAW dispersal can be improved by identifying the peak emergence days of FAW investigated in maize fields, which are determined by the development of FAW responding to the surrounding temperature [40]. The most commonly studied factor of the relationship between life-history stage and environment is the effect of air temperature [30]. In this study, the minimum DMSAT in the Yangtze River Valley from 20 April to 30 July in 2014–2018 was much higher than 13.8 °C, which is the lowest temperature threshold for FAW development [40], indicating a suitable temperature in the Yangtze River Valley, where FAW has been successfully surviving and breeding. The emigration dates for the first and second generations of the newly-invaded FAW from the Yangtze River Valley were concentrated from 20 May to 30 July.

Previous studies showed that the long-distance migration pathways of FAW are facilitated by transport on suitable winds and synoptic wind patterns in the nighttime [10,18,47,54]. In this study, we employed a unique trajectory analysis modeling approach by linking the flight characteristics and development of FAW to the meteorological data from the past five years. This approach has been validated and widely deployed in simulating trajectories of migratory insect pests worldwide [36,44], especially of moths including FAW [27,28,55]. The FAW migration routes we identified are encouragingly accurate, and our analysis thus underlines the role of nocturnal wind patterns on the studied nights in the emigration process and subsequent infestation of FAW populations in the Yangtze River Valley. The development of the summer monsoon over East China through May to July produces highly favorable winds [28,36,56] for carrying the winged FAW from the Yangtze River Valley northwards into Northern China, the Korean Peninsula and Japan. However, windborne insects en route at high altitudes can become concentrated and forced to descend or land because of unfavorable atmospheric factors [36]. In the present study, we considered a lower temperature threshold for the development and flight behaviors of FAW; however, other abiotic factors or phenomena, such as strong downdrafts and rainfall [36,57], which affect insect flight initiation and termination, were not incorporated in the current analysis because the mechanisms specific for FAW moths have not been clarified. Additional analyses are therefore still needed to fully understand the effects of meteorological cues on the migration process of this invasive pest, and these insights will lead to the creation and introduction of better monitoring, forecasting, and control strategies.

Our results also identified that the Korean Peninsula (15,178 endpoints of the total landing in South Korea, 8661 endpoints falling into North Korea) and Japan (2352 endpoints) would be infested by the first generation of the newly-invaded FAW originating from the Yangtze River Valley as early as during 5–7 June, but before 30 June, which is in good agreement with occurrences of invasive caterpillars of FAW monitored in these countries [58,59]. As reported, 1–3 instar FAW larvae in South Korea were first detected on 14 June 2019, whereas Japan confirmed the first appearance of FAW on 27 June 2019. Our results suggest that source populations of the earliest FAW invading the Korean Peninsula and Japan mostly originated from HN (Figure 1), in which the newly-invaded population was investigated from 1–10 May 2019. In addition to HN, GD, located in the tropical and southern subtropical zones (comprising YN, GD, GX and HI, Figure 1), can also provide migrants to colonize the Korean Peninsula if the FAW conducts more than two successive night flights [28]. Transoceanic migrations of insects were frequently reported [60], and strong flyers, such as moths and butterflies, can even complete two-to-three-day journeys by crossing the 2000 km of ocean between Australia and New Zealand [61]. Moth travelers surviving during migrations over the Bohai Sea of China have also been commonly recorded [62]. As anticipated, FAW moths have crossed the Mozambique Channel that has a maximum width of 419 km to breed in Madagascar [63]. These pioneering works and the firstly-observed occurrences of FAW in South Korea and Japan provide strong evidence that FAW populations from the Yangtze River Valley are able to migrate long distances across the sea and possibly are the source of FAW colonizing these two neighboring countries.

Previous analyses emphasized that FAW populations from tropical and southern subtropical zones of China have been continuously immigrating into the Yangtze River Valley from March to August [28,55]. Myanmar is one of the key source regions of FAW immigrants in YN [27]. Indeed in YN, FAW generations are present throughout the coldest part of the winter (January), wherever the host plants are available [27,28]. Our results could further suggest that the possibility of population communication between the Yangtze River Valley and the tropical and southern subtropical zones populations is relatively high when the winds blow toward the south or southwest at night. Briefly, substantial mixing of the migrants would occur among the populations of the tropical and southern subtropical zones followed by a southward return, and FAW is establishing itself as one of the major pests in China. Therefore, further studies should be carried out to identify impacts of non-native invasive FAW on agricultural production, biodiversity and ecosystem function.

## 5. Conclusions

Our extensive study of migration patterns and biometeorological processes elucidated the population dynamics of newly-invaded FAW in the Yangtze River Valley, and we confirmed the importance of the Yangtze River Valley as the source of migrants colonizing Northern China during May to July. We proposed that the migration of FAW between the Yangtze River Valley (i.e., middle and northern subtropical zones of China) and the tropical and southern subtropical zones of China would form a circuit due to the advance and retreat of the prevailing southerly winds. Simulation and prediction of migratory insect pests such as FAW, which are too small to be directly tracked, remains challenging. Our data provide a basis for monitoring and early warning of FAW emigrating from the Yangtze River Valley of China. Further work is required to monitor and quantify the migration magnitude, combined with an atmospheric trajectory model, systematic field investigation of populations and long-term-operated radars, which would increase our predictive capabilities relating to migratory pests.
