# Correction: Wu, Q.-L.; et al. Estimation of the Potential Infestation Area of Newly-Invaded Fall Armyworm *Spodoptera Frugiperda* in the Yangtze River Valley of China. *Insects* 2019, *10*, 298

**DOI:** 10.3390/insects11080521

**Published:** 2020-08-11

**Authors:** Qiu-Lin Wu, Li-Mei He, Xiu-Jing Shen, Yu-Ying Jiang, Jie Liu, Gao Hu, Kong-Ming Wu

**Affiliations:** 1 State Key Laboratory for Biology of Plant Diseases and Insect Pests, Institute of Plant Protection, Chinese Academy of Agricultural Sciences, Beijing 100193, China; wuqiulin89@126.com (Q.-L.W.); helimei91@163.com (L.-M.H.); shenxj1230@163.com (X.-J.S.); 2 College of Plant Protection, Fujian Agriculture and Forestry University, Fuzhou 350002, China; 3 National Agro-Tech Extension and Service Center, Beijing 100125, China; jiangyujing@agri.gov.cn (Y.-Y.J.); cbliujie@agri.gov.cn (J.L.); 4 Department of Entomology, Nanjing Agricultural University, Nanjing 210095, China; hugao@njau.edu.cn

The authors wish to make the following correction to this paper [1]:

We found that one sentence is repeatedly printed in the results Section 3.4, lines 2–5. The sentence should read: “In particular, the estimated first generation of newly-invaded FAW emerged on 20–30 May for A1, on 1–10 June for B1, on 11–20 June for C1, and on 21–30 June for D1, whereas the peak adult emergence of the second generation was suggested to occur on 10–20 July for A2, 1–10 July for B2, 11–20 July for C2, and 21–30 July for D2, respectively.”

The authors would like to apologize for any inconvenience caused to the readers by these changes.

## References

[1] Wu Q.-L., He L.-M., Shen X.-J., Jiang Y.-Y., Liu J., Hu G., Wu K.-M. (2019). Estimation of the potential infestation area of newly-invaded fall armyworm *Spodoptera frugiperda* in the Yangtze River Valley of China. Insects.

